# Joint Estimation of Coherent Signal DOA and Polarization Parameters Based on Improved BSBL

**DOI:** 10.3390/s26123818

**Published:** 2026-06-16

**Authors:** Yan Du, Jun Peng, Dacheng Hu, Junhui Wang, Hao Zeng

**Affiliations:** 1 The 10th Research Institute of China Electronics Technology Group Corporation, Chengdu 610036, China; bella_1021_yi@sina.com (Y.D.); hdc1988422@sina.com (D.H.); xidian_wjh@163.com (J.W.); 2 School of Microelectronics and Communication Engineering, Chongqing University, Chongqing 401331, China; junp@stu.cqu.edu.cn

**Keywords:** coherent signal, polarization estimation, DOA estimation, BSBL

## Abstract

This paper introduces a method for estimating signal direction of arrival (DOA) and polarization parameters with coherent signal sources based on an improved block structure of block sparse Bayesian learning (BSBL). The array is a uniform linear dual-polarization array composed of doubly fed orthogonal linearly polarized elements and is used to receive the horizontal electric field component and the vertical electric field component. By constructing a decoupled block sparse Bayesian model, the parameter estimation problem is transformed into a sparse recovery problem, and the improved block structure is used to achieve joint estimation of the DOA and polarization parameters in the complex domain within the sparse Bayesian framework. The estimation method enhances the signal discrimination ability in multiple near-angle source incidence scenarios, its parameter estimation performance is superior under the condition of small sampling, and the excellent performance of the algorithm is proved by the analysis and simulation of the estimation error.

## 1. Introduction

Previous research methods mostly focused on parameter estimation of multiple incoherent signal sources. However, in the actual electronic reconnaissance environment, highly coherent signals formed by multipath effects are more common. Under the condition of highly coherent signal sources, the covariance matrix of the array’s received data shows a rank deficiency phenomenon, resulting in most signal subspace class methods being no longer applicable.

The spatial smoothing (SS) algorithm, through subarray division, selects the subarray with the same array structure to receive data for the averaging operation of the covariance matrix, which can restore the rank of the covariance matrix [1]. However, this method will lose the aperture of the array and sacrifice the degrees of freedom of the array, resulting in poor estimation performance of the algorithm. The polarization smoothing (PS) algorithm divides sub-arrays based on the data received by the same components of each polarization-sensitive array element and then calculates their covariance matrices [2,3]. Different weighting matrices are used to smooth these covariance matrices [4,5,6]. The polarization domain smoothing method can effectively achieve de-coherence without reducing the effective aperture of the overall array. Especially when the multiple angles of spatial incidence are relatively small, its performance is superior to that of the spatial smoothing algorithm. However, the ability to de-coherence is limited by the number of polarization-sensitive components.

Parameter estimation algorithms based on sparse reconstruction classes perform better than Bayesian learning algorithms in the case of small snapshots, and they possess inherent coherence-reducing capabilities [7]. The authors of [8] conducted research on spatial grid partitioning and DOA estimation methods based on the idea of sparse reconstruction. They utilized the norm constraint of signal sparsity and constructed a sparse representation model for array output data based on the spatial sparsity of incident signals and proposed the classic L1-singular value decomposition (SVD) algorithm for DOA estimation. However, this algorithm uses SVD to extract the signal subspace, so it requires prior knowledge of the number of signal sources; otherwise, it leads to a decline in algorithm performance. The paper [9] proposed a BSBL reconstruction algorithm based on sparse Bayesian learning, discussed the intra-block and inter-block correlations of sparse signals, explained that the BSBL is equivalent to a weighted L1 norm optimization algorithm, and proved that the BSBL is superior to the orthogonal matching pursuit (OMP) algorithm. The paper [10] combined the polarization-sensitive array signal model with the block sparse recovery model, based on the distributed polarization-sensitive array model and proposed to vectorize multi-vector snapshot data to achieve joint polarization–DOA estimation. The sparse reconstruction class algorithms mentioned above can effectively estimate the parameters of coherent sources and also have better adaptability, precise sparse recovery, and noise robustness within the Bayesian framework. The paper [11] presents an iterative Variational Bayes (VB) algorithm that allows sparse recovery of the desired transmitted vector. The VB algorithm is derived based on the latent variables introduced in the Bayesian model in hand and has a significant advantage when the distances between the source points are relatively close. However, these methods are all based on real-number domain sparse reconstruction algorithms and do not incorporate sparse reconstruction models in the complex domain. Oraintara et al. presented a sparse Bayesian learning model based on the complex number domain, which can achieve the reconstruction of sparse signals well. However, the convergence of the hyperparameters of this model is poor. Especially in low signal-to-noise ratio scenarios, the estimation of the posterior distribution is prone to instability, resulting in reconstruction failure [12]. Oliveri considered dividing the complex numbers into the real part and the imaginary part and then using real number Bayesian learning algorithms to reconstruct the sparse signals separately. However, the drawback of this algorithm is that the computational complexity of processing the real part and the imaginary part separately increases exponentially, and the algorithm complexity also rises [13].

In this paper, we address these limitations. We propose an improved block structure coherent cancellation algorithm based on BSBL, which can estimate DOA and polarization parameters of the coherent signal. Compared with DOA and polarization parameter estimation methods mentioned above, the proposed algorithm has the following advantages: (1) It enhances the signal discrimination ability in multiple near-angle source incidence scenarios. (2) It not only can estimate the polarization parameters of multiple coherent signals but also can simultaneously estimate the direction of arrival of multiple coherent signals. (3) Its parameter estimation performance is superior under the condition of small sampling.

## 2. Signal Model

**Figure 1:**
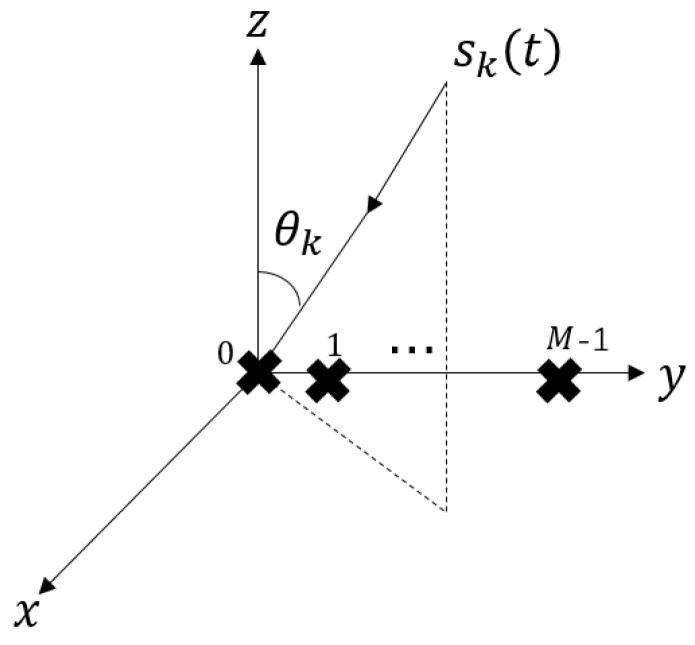
Schematic diagram of uniform orthogonal dual-polarized linear array.

We start by considering the uniform linear array with sensors at a number of *M* different locations in the *x*-axis. Each array element is a dual-feed orthogonal linear polarization unit, as shown in Figure 1. Adjacent ones are supposed to be mutually half wavelength spacing, and the signal wavelength is λ.

Suppose there is a single antenna at the zero point of a spherical coordinate system, facing up to the polarization of upcoming signals. Suppose there are *K* coherent electromagnetic wave signals entering the space, and the *k*th signal is assumed to arrive from direction $\theta_{k}$, where $\theta_{k}$ stand for the elevation angle. Let the wave be a transverse electromagnetic wave, and consider the polarization ellipse stimulated by electric field in a constant transverse plane. Polarization parameters are denoted as $\gamma_{k}$ and $\eta_{k}$.

Then the spatial orientation vector $a(\theta_{k})$ of the *k*th signal is expressed as

(1) $$a\left(\theta_{k}\right)={\left[1,e^{-j\psi_{k}},\ldots ,e^{-j\left(M-1\right)\psi_{k}}\right]}^{T}$$

where *d* is the spacing between array elements, $\psi_{k}=2\pi d\sin\theta_{k}/\lambda$.

And the polarization orientation vector of the signal $a_{p}\left(\theta_{k},\gamma_{k},\eta_{k}\right)$ is

(2) $$a_{p}\left(\theta_{k},\gamma_{k},\eta_{k}\right)=\underset{≜\Theta (\theta_{k})}{\underbrace{\left[\begin{array}{cc} -1 & 0\\ 0 & cos\theta_{k} \end{array}\right]}}\underset{≜g(\gamma_{k},\eta_{k})}{\underbrace{\left[\begin{array}{c} cos\gamma_{k}\\ sin\gamma_{k}\cdot e^{j\eta_{k}} \end{array}\right]}}$$

Then it can be known that the output signal of the polarization array at time *t* is

(3) $$x\left(t\right)=\sum_{k=1}^{K}a\left(\theta_{k}\right)⊗a_{p}\left(\theta_{k},\gamma_{k},\eta_{k}\right)s_{k}\left(t\right)+n\left(t\right)$$

where $x\left(t\right)\in C^{2M\times 1}$ represents the vector of array output signals, ⊗ denotes the Kronecker product, $s_{k}\left(t\right)$ is the incident signal waveform, and n(t) is Gaussian white noise.

From (3), it can be seen that the DOA of the signal and the polarization parameters are coupled in the joint steering matrix. Considering the complete dictionary construction method of the sparse Bayesian recovery class algorithm, and in order to enhance the signal discrimination ability in multiple near-angle source incidence scenarios, this paper based on the properties of the Kronecker product decouples the DOA and the polarization parameters in the joint steering matrix as follows:

(4) $$\begin{array}{cc} & x\left(t\right)=\sum_{k=1}^{K}\left(a_{s}\left(\theta_{k}\right)⊗a_{p}\left(\theta_{k},\gamma_{k},\eta_{k}\right)\right)s_{k}\left(t\right)+n\left(t\right)\\ & =\sum_{k=1}^{K}\left(a_{s}\left(\theta_{\kappa }\right)⊗\left(\Theta \left(\theta_{k}\right)g\left(\gamma_{k},\eta_{k}\right)\right)\right)s_{k}\left(t\right)+n\left(t\right)\\ & =\sum_{k=1}^{K}\left(\left(a_{s}\left(\theta_{k}\right)⊗\Theta \left(\theta_{k}\right)\right)\left(I_{1\times 1}⊗g\left(\gamma_{k},\eta_{k}\right)\right)\right)s_{k}\left(t\right)+n\left(t\right)\\ & =\sum_{k=1}^{K}\left(a_{s}\left(\theta_{k}\right)⊗\Theta \left(\theta_{k}\right)\right)\left(g\left(\gamma_{k},\eta_{k}\right)s_{k}\left(t\right)\right)+n\left(t\right)\\ & =A^{\prime }s^{\prime }\left(t\right)+n\left(t\right) \end{array}$$

where $A^{\prime }$ is an array joint steering matrix that only contains signal angle information, and $s^{\prime }\left(t\right)$ is the incident signal vector that includes signal polarization information. I is a unit matrix. Therefore, by utilizing the property of the Kronecker product, the DOA and polarization parameters are separated in the joint steering vector, achieving parameter decoupling. With Kronecker product decomposition, DOA and polarization information are fully decoupled: the overcomplete dictionary only bears angular information, whereas polarization features are encapsulated inside sparse signal blocks. This dramatically reduces dictionary redundancy and mutual coherence compared with conventional dictionaries containing coupled angle-polarization information simultaneously.

**Figure 2:**
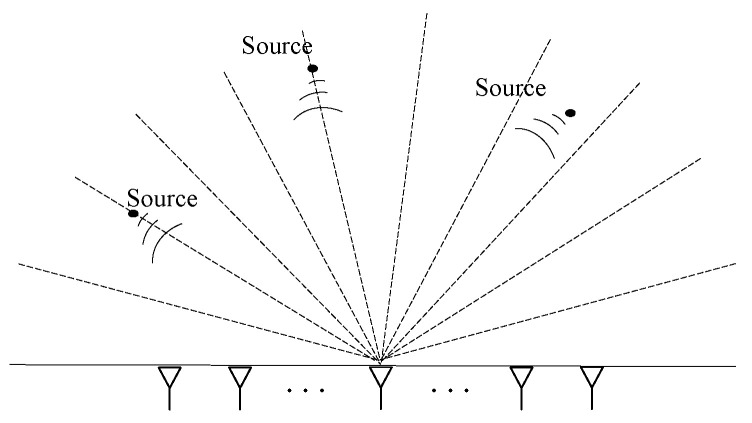
Schematic diagram of array spatial grid division.

Based on the sparse Bayesian recovery algorithm concept, a sparse signal is constructed by utilizing the spatial sparsity of the incident signal. At the same time, considering the limited nature of the incident signal in the spatial domain, within a certain quantization error range, the continuous angular set in the spatial domain is divided into a finite discrete angular set by using a grid. The exhaustive method is adopted to obtain an over-complete redundant discrete angular set $\bar{\theta }=\left[{\bar{\theta }}_{1},{\bar{\theta }}_{2},\ldots ,{\bar{\theta }}_{\bar{K}}\right]$, where $\bar{K}$ represents the number of grid points. This paper considers a linear array. For the sake of generality, the grid interval r=1° is used to uniformly divide the spatial range of [−90°,90°] into a grid, and the specific grid division schematic diagram is shown in Figure 2.

The dotted lines in Figure 2 represent the grid points for spatial division, and the solid circles indicate the direction of the incident signal. From the figure, it can be seen that some of the incident signals fall precisely on the grid points without deviation, while some do not fall precisely on the grid points. This reflects a certain quantization error brought about by the application of grid division. This paper analyzes the simplest sparse reconstruction model, which assumes that all incident signal directions are precisely located on the discrete grid points. Therefore, Equation (4) can be rewritten as

(5) $$x\left(t\right)={\bar{A}}^{\prime }{\bar{s}}^{\prime }\left(t\right)+n\left(t\right)$$

where $\bar{A}$ is the super-complete redundant array joint steering matrix dictionary corresponding to the super-complete redundant angle set $\bar{\theta }$, and it only contains the signal angle information; ${\bar{s}}^{\prime }\left(t\right)$ is the spatial sparse signal vector composed of the incident signals that contain the signal polarization information, and its definition is

(6) $${\bar{s}}_{k}^{\prime }\left(t\right)=\left\{\begin{array}{cc} {\bar{s}}_{\bar{k}}^{\prime }\left(t\right)=s_{k}^{\prime }\left(t\right), & {\bar{\theta }}_{\bar{k}}=\theta_{k}\\ 0 & others \end{array}\right.,\bar{k}=1,2,\ldots \bar{K},k=1,2,\ldots K$$

**Figure 3:**
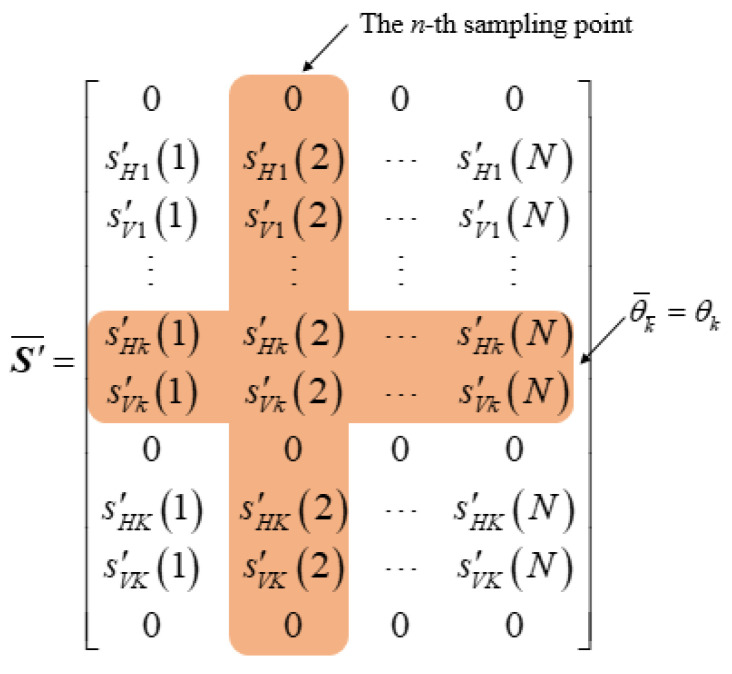
Structured sparse signals in the airspace.

Since $K<M\ll \bar{K}$, for *K* incoming signals, ${\bar{s}}^{\prime }\left(t\right)$ is a sparse matrix with a sparsity of 2K rows. Extending (5) to the multi-sweep scenario, the array receives the multi-sweep sampled data signal model can be expressed as

(7) $$X={\bar{A}}^{\prime }{\bar{S}}^{\prime }+N$$

where $X\in C^{2M\times N}$ represents the received signal of the array, and *N* is the number of sampling snapshots. ${\bar{S}}^{\prime }\in C^{2\bar{K}\times N}$ is a spatially sparse signal composed of *N* sampling points, with the structured sparsity as shown in Figure 3. $N\in C^{2M\times N}$ is a Gaussian white noise signal.

As can be seen from Figure 3, in the sparse signal ${\bar{S}}^{\prime }$, each incident signal has a corresponding horizontal polarization component and a vertical polarization component, which are related through the polarization parameters of the signal and have a relationship as shown in (8).

(8) $$\frac{s_{Hk}^{\prime }\left(n\right)}{s_{Vk}^{\prime }\left(n\right)}=\tan\gamma_{k}e^{j\eta_{k}}$$

where $s_{Hk}^{\prime }\left(n\right)$ and $s_{Vk}^{\prime }\left(n\right)$ individually represent the horizontal polarization component and the vertical polarization component of the kth signal at the nth sampling instant.

At the same time, for the same signal, there is also temporal correlation between each sampling snapshot. The signal parameter estimation algorithm based on BSBL in this paper utilizes these relationships between the signals to structurally divide the received signal into blocks, with each block corresponding to an incident signal, and each block contains all the sampling points of the horizontal and vertical polarization components of the received signal. This block division method not only retains all the information of the same signal source but also makes the block structure the smallest unit of the sparse signal, laying the foundation for subsequent improvements to the block structure.

Equation (7) represents the multi-measurement vector (MMV) signal model under the condition of array multi-shot sampling data. To adapt to the BSBL algorithm framework, the MMV signal model needs to be transformed into an SMV signal model. That is, (7) undergoes a vectorization operation to obtain

(9) x=Φs+n

where $x=vec\left(X^{T}\right)\in C^{2MN\times 1}$ represents the received signal vector, $\Phi ={\bar{A}}^{\prime }⊗I_{N}\in C^{2MN\times 2\bar{K}N}$ is the identity matrix, $s=vec\left(\;{\bar{S}}^{\prime T}\right)\in C^{2\bar{K}N\times 1}$ represents the incident signal vector, $n=\nu ec\left(N^{T}\right)\in C^{2MN\times 1}$ represents the noise signal vector, and vec(·) indicates the operation of vectorizing the matrix, converting the matrix representation into a vector form.

For the received data, based on the structure of the sparse signal ${\bar{S}}^{\prime }$ shown in Figure 3, the 2N sampling data in each received data can be divided into one block. The block structure is as follows:

(10) $$s=\left[\underset{s_{1}}{\underbrace{s_{1},\ldots ,s_{2N}}},\underset{s_{2}}{\underbrace{s_{2N+1},\ldots ,s_{4N}}}\ldots \underset{s_{\bar{K}}}{\underbrace{s_{2N(\bar{K}-1)+1},\ldots ,s_{2\bar{K}N}}}\right]$$

where $s_{\bar{k}}$ represents the *i*th block, $k=1,\ldots ,\bar{K}$, and the length of each block is 2N. Among these $\bar{K}$ blocks, only $k\left(k<\bar{K}\right)$ blocks are non-zero, and the positions of the non-zero blocks are unknown.

This is called the standard block sparse model. The customized block structure restricts signal sparsity at block level rather than single-element sparsity, further reducing the dictionary condition number by constraining the correlation range of dictionary columns. From a mathematical perspective, this block sparsity can reduce the search freedom in the signal space, significantly improving the reconstruction accuracy and robustness of the algorithm and achieving better recovery performance. From the perspective of signal processing, considering the time correlation of each signal and the structural correlation between the polarization components, using this block structure can further enhance the sparse recovery performance [14].

## 3. Algorithm Design

### 3.1. BSBL Algorithm Framework

For a signal with block sparsity characteristics, in order to avoid over-matching, the sparse Bayesian learning provides the prior information of the signal and imposes constraints on the signal to be estimated, which enables the signal to more easily satisfy the sparsity condition and is conducive to the high-precision restoration of the sparse reconstruction algorithm [15]. Assume that the $\bar{k}$th block $s_{\bar{k}}$ follows a zero-mean Gaussian distribution as

(11) $$p\left(s_{\bar{k}}\right)∼N\left(0,\alpha_{\bar{k}}B_{\bar{k}}\right),\;\bar{k}=1,2,\ldots ,\bar{K}$$

where $\alpha_{k}$ represents the block sparsity of the signal and is a non-negative parameter that determines whether the obtained result is a signal or noise. When $\alpha_{k}$ approaches 0, it indicates a higher possibility that this signal block is noise, and the corresponding signal block will be set to a zero vector. $B_{k}$ is a positive definite matrix that controls the correlation structure information within the signal block. Assuming that the noise vector also follows a zero-mean Gaussian distribution p(n)∼N(0,λI), λ is the variance of the noise, and usually it is unknown. Then the prior probability distribution of the signal s satisfies $p\left(s\right)∼N\left(0,\Sigma_{0}\right)$, where $\Sigma_{0}$ is a block diagonal matrix that satisfies

(12) $$\Sigma_{0}=\mathrm{diag}\left\{\alpha_{1}B_{1},\ldots ,\alpha_{\bar{K}}B_{\bar{K}}\right\}$$

Therefore, the observation vector follows a Gaussian distribution with a mean of Φs and a variance of λ, and its expression is

(13) p(x|s;λ)∼N(Φs,λI)

In the SBL method, there are generally two parameter estimation methods, namely, Type I estimation and Type II estimation. In Type I estimation, more emphasis is placed on signal z rather than the entire posterior distribution. This point estimate value is called the maximum a posteriori probability estimate, while in Type II estimation, more attention is paid to the influence of signal variance or accuracy, and Type II estimation requires approximating the posterior distribution, which is called maximum likelihood estimation [16]. Reference [17] provides a detailed explanation of the differences between Type I estimation and Type II estimation, and concludes that the reconstruction performance of Type II estimation is superior to that of Type I estimation. Therefore, in this section, Type II estimation is adopted to estimate the posterior probability density function and likelihood function of s. Based on the above assumptions and prior information, according to Bayes’ theorem, the posterior distribution of signal s can be obtained as

(14) $$p\left(s|x;\lambda ,{\left\{\alpha_{\bar{k}},B_{\bar{k}}\right\}}_{\bar{k}=1}^{\bar{K}}\right)∼N\left(\mu_{s},\Sigma_{s}\right)$$

where

(15) $$\left\{\begin{array}{c} \mu_{s}=\sum_{0}\Phi^{T}{\left(\lambda I+\Phi \sum_{0}\Phi^{T}\right)}^{-1}x\\ \Sigma_{s}={\left(\sum_{0}^{-1}+\frac{1}{\lambda }\Phi^{T}\Phi \right)}^{-1}. \end{array}\right.$$

Then the posterior probability estimate of s can be written as

(16) $$\hat{s}≜\mu_{s}=\sum_{0}\Phi^{T}{\left(\lambda I+\Phi \sum_{0}\Phi^{T}\right)}^{-1}x$$

When the mean value reaches its maximum, $\hat{s}$ becomes the recovered signal. In the posterior probability density function, if the values of parameters such as $\lambda ,\alpha_{\bar{k}},B_{\bar{k}}$ are obtained through iterative processes, the reconstructed signal can be obtained. Let the parameters obtained from the posterior probability density function be represented by $∆=\{\lambda ,\alpha_{\bar{k}},B_{\bar{k}}\}$. According to the likelihood function of Type II estimation, ∆ can be expressed as

(17) $$L\left(∆\right)=\log|\lambda I+\Phi \Sigma_{0}\Phi^{T}|+x^{T}{\left(\lambda I+\Phi \Sigma_{0}\Phi^{T}\right)}^{-1}x$$

Then the cost function equation of ∆ is

(18) $$\begin{array}{cc} & Q\left(∆\right)=E_{s|x,{∆}^{P}}\left[\log p\left(x,s,∆\right)\right]\\ & =E_{s|x;{∆}^{P}}\left[\log p\left(x|s;\lambda \right)\right]+E_{s|x;{∆}^{p}}\left[\log p\left(s;\left\{\alpha_{\bar{k}},B_{\bar{k}}\right\}\right)\right]\\ & =Q\left(\lambda \right)+Q\left(\alpha_{\bar{k}},B_{\bar{k}}\right) \end{array}$$

where ${∆}^{P}$ represents the value of ∆ from the previous iteration. Expanding $Q\left(\alpha_{\bar{k}},B_{\bar{k}}\right)$, we can obtain

(19) $$\begin{array}{cc} & Q\left(\alpha_{\bar{k}},B_{\bar{k}}\right)\propto -\frac{N}{2}\log\left(|\Gamma |\right)-\frac{\bar{K}}{2}log\left(|B|\right)\\ & -\frac{1}{2}Tr\left[\left(\Gamma^{-1}⊗B^{-1}\right)(\Sigma_{s}+\mu_{s}\mu_{s}^{T}\right)] \end{array}$$

where $\Gamma ≜diag\left(\alpha_{1},\alpha_{2},\ldots ,\alpha_{\bar{K}}\right)$ is a diagonal matrix. By using the gradient method to take the partial derivative of $\alpha_{\bar{k}}$, we can obtain

(20) $$\left\{\begin{array}{c} \frac{\partial Q}{\partial \alpha_{\bar{k}}}=-\frac{N}{\alpha_{\bar{k}}}+\frac{1}{2\alpha_{\bar{k}}^{2}}Tr\left[B_{\bar{k}}^{-1}\left(\Sigma_{s}^{\bar{k}}+\mu_{s}^{\bar{k}}{\left(\mu_{s}^{\bar{k}}\right)}^{T}\right)\right]\\ \alpha_{\bar{k}}\leftarrow \frac{Tr\left[B_{\bar{k}}^{-1}\left(\Sigma_{s}^{\bar{k}}+\mu_{s}^{\bar{k}}{\left(\mu_{s}^{\bar{k}}\right)}^{T}\right)\right]}{2N},\;\bar{k}=1,2,\ldots ,\bar{K} \end{array}\right.$$

Similarly, taking the partial derivative with respect to parameter $B_{\bar{k}}$ will obtain

(21) $$\left\{\begin{array}{c} \frac{\partial Q}{\partial B_{\bar{k}}}=-\frac{\bar{K}}{2}\;B_{\bar{k}}^{-1}+\frac{1}{2}\sum_{\bar{k}=1}^{\bar{K}}\frac{1}{\gamma_{\bar{k}}}B_{\bar{k}}^{-1}\left(\Sigma_{s}^{\bar{k}}+\mu_{s}^{\bar{k}}{\left(\mu_{s}^{\bar{k}}\right)}^{T}\right)B_{\bar{k}}^{-1}\\ B_{\bar{k}}\leftarrow \frac{1}{\bar{K}}\sum_{\bar{k}=1}^{\bar{K}}\frac{\Sigma_{s}^{\bar{k}}+\mu_{s}^{\bar{k}}{\left(\mu_{s}^{\bar{k}}\right)}^{T}}{\gamma_{\bar{k}}} \end{array}\right.$$

Taking the partial derivative of Q(λ) with respect to λ simultaneously can obtain

(22) $$\left\{\begin{array}{c} Q\left(\lambda \right)=-M\mathrm{Nlog}\lambda -\frac{\left[\|x-\Phi \mu_{s}{∥}_{2}^{2}+\lambda \left[2\bar{K}N-Tr\left(\Sigma_{s}\Sigma_{0}^{-1}\right)\right]\right]}{2\lambda }\\ \lambda \leftarrow \frac{\|x-\Phi \mu_{s}{∥}_{2}^{2}+\lambda \left[2\bar{K}N-Tr\left(\Sigma_{s}\Sigma_{0}^{-1}\right)\right]}{4MN} \end{array}\right.$$

The reconstruction algorithm based on BSBL is prone to cause overfitting problems. However, if matrix B is a Toeplitz positive definite matrix and $B_{\bar{k}}=B\left(\forall \bar{k}\right)$ can effectively prevent overfitting [9], then the overfitting phenomenon can be effectively avoided as

(23) $$B=Toeplitz\left(\left[\begin{array}{c} 1,b,\ldots b^{d-1} \end{array}\right]\right)=\left[\begin{array}{cccc} 1 & b & \ldots & b^{d-1}\\ \vdots & \vdots & \vdots & \vdots \\ b^{d-1} & b^{d-2} & \ldots & 1 \end{array}\right]$$

where *d* is the size of each block, $b=m_{1}/m_{0}$, while the values of $m_{1}$ and $m_{0}$ are the mean values of the main diagonal elements and the secondary diagonal elements in matrix B, respectively.

Thus, the estimated value of parameters $\lambda ,\alpha_{\bar{k}},B_{\bar{k}}$ in the posterior probability density function of signal s has been obtained. Then, by substituting these parameter estimates into (15) for iteration, the estimated values of mean $\mu_{s}$ and $\Sigma_{s}$ can be obtained.

### 3.2. Improved Block Structure

First, the plural model is divided into the real part and the imaginary part, $x=x_{R}+jx_{I}$, $\Phi =\Phi_{R}+j\Phi_{I}$, $s=s_{R}+js_{I}$, $n=n_{R}+jn_{I}$. Then (9) is transformed into a complex block sparse recovery model as

(24) $$\left[\begin{array}{c} x_{R}\\ x_{I} \end{array}\right]=\left[\begin{array}{cc} \Phi_{R} & -\Phi_{I}\\ \Phi_{I} & \Phi_{R} \end{array}\right]\left[\begin{array}{c} s_{R}\\ s_{I} \end{array}\right]+\left[\begin{array}{c} n_{R}\\ n_{I} \end{array}\right]$$

The size of each block is 2N×1, corresponding to the real or imaginary part of the horizontal polarization component and the vertical polarization component. In the above equation, the non-zero terms of $s_{R}$ in the recovered signal, and the non-zero terms of $s_{I}$, all correspond to the DOA estimation of the signal. However, due to the influence of noise, the DOA of the real and imaginary parts may not align, and a parameter pairing operation needs to be performed with the polarization parameters. In addition, this operation of separating the real and imaginary parts may disrupt the polarization information structure of the signal, resulting in the inability to accurately recover it and thus unable to estimate the polarization parameters.

**Figure 4:**
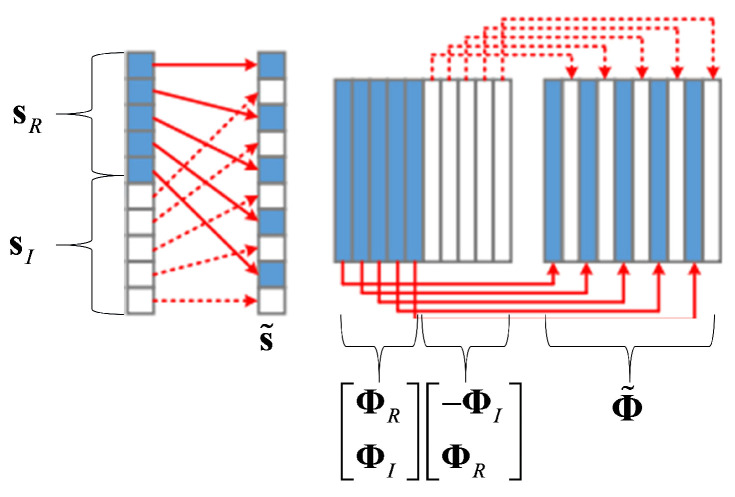
Schematic diagram of the improved block structure.

The reason for the above problem is that the same signal is divided into the real part and the imaginary part in the signal to be restored and is processed separately. For the BSBL algorithm, this means that the estimation of the real part and the imaginary part is not constrained at the same position. Therefore, in this paper, the DOA of the real part and the imaginary part of the same signal are constrained at the same position so as to avoid the above problem. Based on this idea, the transformation of (27) is shown in Figure 4.

For each block $s_{\bar{k}}\left(\bar{k}=1,2,\ldots ,\bar{K}\right)$, the following transformation is carried out:

(25) $${\tilde{s}}_{\bar{k}}=P\cdot {\left[\begin{array}{c} Re\left(s_{\bar{k}}\right)\\ Im\left(s_{\bar{k}}\right) \end{array}\right]}_{4N\times 1}$$

(26) $$\tilde{\Phi }=ivec{\left(vec{\left(\left[\begin{array}{c} \Phi_{R}\\ \Phi_{I}\\ -\Phi_{I}\\ \Phi_{R} \end{array}\right]\right)}^{T}\right)}_{4MN\times 4\;\vec{K}N}$$

where $P\in R^{4\;N\times 4\;N}$ is a permutation matrix, and its specific expression form is

(27) $$P\left(i,j\right)=\left\{\begin{array}{cc} 1, & i=2n-1,\;j=n\\ 1, & i=2n,\;j=2N+n\\ 0, & others \end{array}\right.$$

where n=1,2,…,2N, ivec(·) performs matrix operations on column vectors, indicating the transformation of column vectors into matrices of dimension 4MN×4KN.

Through this transformation, a new block sparse recovery model can be obtained as

(28) $$\tilde{x}=\tilde{\Phi }\tilde{s}+\tilde{n}$$

Each incident signal corresponds to a block of size 4N, which respectively represents the real and imaginary parts of the horizontal polarization component, and the real and imaginary parts of the vertical polarization component. The newly designed 4N-dimensional unified block constraint bundles real/imaginary terms of horizontal/vertical polarization within an identical block from the Bayesian prior setup, inherently eliminating real-imaginary mismatch without extra post-matching operation. Based on (28) and the framework of the block sparse recovery algorithm, the DOA estimation and polarization parameter estimation of the signal can be completed.

From (16), it can be seen that the estimated value of the mean $\mu_{s}$ is the recovered signal $\tilde{s}={\left[\begin{array}{cc} {\tilde{s}}_{R} & {\tilde{s}}_{I} \end{array}\right]}^{T}\in R^{4\bar{K}N\times 1}$, and the result $\hat{s}={\tilde{s}}_{R}+j{\tilde{s}}_{I}$ is calculated. The vector $\hat{s}\in C^{2\bar{K}N\times 1}$ is transformed into the matrix $\hat{S}\in C^{2\bar{K}\times N}$. In the recovered signal matrix $\hat{S}$, each pair of adjacent rows form a block, corresponding to the ultra-complete redundant discrete angle set $\bar{\theta }$. And each two adjacent non-zero rows correspond to a signal block. Under ideal conditions, the angle corresponding to the positions of each pair of adjacent non-zero rows in the discrete angle set $\bar{\theta }$ in the signal matrix $\hat{S}$ is the estimated value of the incident signal DOA. However, due to the presence of noise, many components of the reconstructed sparse vector have very small amplitudes. Therefore, performing spatial spectral estimation on the recovered signal matrix $\hat{S}$, the power of each block is calculated as follows:

(29) $$P\left(\bar{k}\right)={\left|\hat{S}\left(2\bar{k}-1:2\bar{k}\right)\right|}^{2}\;\bar{k}=1,2,\ldots ,\bar{K}$$

The mapping angle value of the index $\bar{k}$, which corresponds to the peak values of the largest *K* blocks in the discrete angle set $\bar{\theta }$, is the estimated value ${\hat{\theta }}_{k}$ of the true incident signal DOA, that is

(30) $${\hat{\theta }}_{k}=\bar{\theta }\left(\bar{k}\right)\;k=1,2,\ldots ,K,\bar{k}=1,2,\ldots ,\bar{K}$$

Meanwhile, for each estimated signal block, based on the block structure information given by (8), the polarization parameters of each incident signal can be estimated as

(31) $$\left\{\begin{array}{c} {\hat{\gamma }}_{k}=\mathrm{mean}\left(\arctan|\frac{s_{Hk}^{\prime }\left(n\right)}{s_{Vk}^{\prime }\left(n\right)}|\right)\\ {\hat{\eta }}_{k}=\mathrm{mean}\left(\arg\left(\frac{s_{Hk}^{\prime }\left(n\right)}{s_{Vk}^{\prime }\left(n\right)}\right)\right) \end{array}\right.,n=1,2,\ldots ,N,k=1,2,\ldots ,K$$

where mean(·) is calculating the mean value.

### 3.3. Analysis of Algorithm Complexity

Assume that the number of array elements is *M*, the number of signal sources is *K*, the number of snapshots is *N*, the number of discrete divisions of the spatial domain grid is $\bar{K}$, and the number of BSBL iterations is *I*. The computational complexities of the FBSS algorithm, the improved PS algorithm, the Toeplitz algorithm, the OMP algorithm, the L1-SVD algorithm, and the improved BSBL algorithm proposed in this paper were analyzed. The comparison of the specific algorithm complexities is shown in Table 1.

In practical applications, *I*, *M*, *K*, and *N* are typically much smaller than $\bar{K}$. The terms related to $\bar{K}$ determine the computational load of the algorithm. From the above analysis, the FBSS algorithm and the improved PS algorithm have similar complexities, mainly influenced by the number of array elements, the number of snapshots, and the number of grid points. The OMP algorithm and the L1-SVD algorithm have a significant increase in computational complexity with high-resolution grids. However, the improved block structure BSBL algorithm in this paper avoids $\bar{K}$ cubic meter scale scale operations and replaces the full-space search with low-dimensional block iterations. Under the same simulation conditions, its complexity is significantly lower than that of the OMP algorithm and the L1-SVD algorithm. Nevertheless, compared to traditional smoothing and matrix reconstruction algorithms, the computational complexity of the BSBL algorithm is still relatively large. However, considering its advantages of small estimation error and super-resolution capability under small snapshot number conditions, this computational complexity is acceptable.

## 4. Simulations

To verify the effectiveness of the proposed algorithm, the proposed coherent signal DOA and polarization parameter estimation based on the improved block structure of the BSBL method was simulated and analyzed.

### 4.1. The Effectiveness of the Improved Block Structure

**Figure 5:**
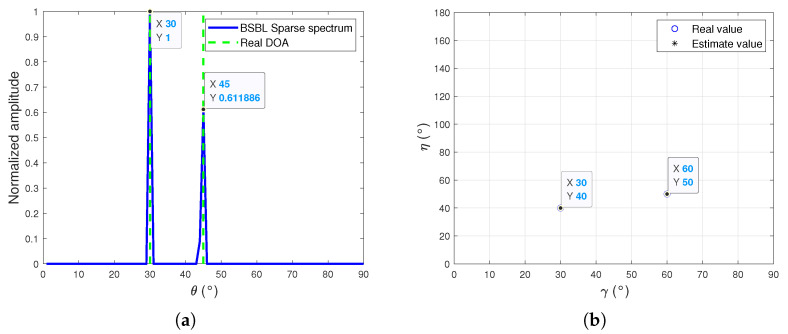
Estimation effect diagrams of DOA parameters and polarization parameters. (**a**) DOA estimation of sparse spectrum. (**b**) Polarization parameter estimation diagram.

There were two narrowband coherent signals in the space. The array element count was M=8, and the spacing between array elements was half a wavelength. Gaussian white noise power was 1. Snapshots N=16, and the signal-to-noise ratio (SNR) was 10 dB, with discrete angle set $\bar{\theta }=\left[1^{\circ }:1^{\circ }:90^{\circ }\right]$ and the grid point number $\bar{K}=90$. The incoming wave directions setting was $\left(\theta_{1},\gamma_{1},\eta_{1}\right)=\left(30^{\circ },30^{\circ },40^{\circ }\right),\left(\theta_{2},\gamma_{2},\eta_{2}\right)=\left(45^{\circ },60^{\circ },50^{\circ }\right)$. The relative complex gain of the coherent signal source was set to 1, $e^{j\pi /4}$ when it was generated. The number of Monte Carlo simulations was 100. The unit of the results is degrees. The specific simulation results are shown in Figure 5.

Figure 5 shows that the improved BSBL algorithm proposed in this paper is capable of accurately distinguishing these two coherent signal sources and estimating the DOA and polarization parameters, and it has solved the problem that the traditional real and imaginary separation algorithm may cause the real and imaginary parts to fail to align or the estimation of the real or imaginary part to be invalid.

### 4.2. The Resolution of the Intervals at Different Angles

**Figure 6:**
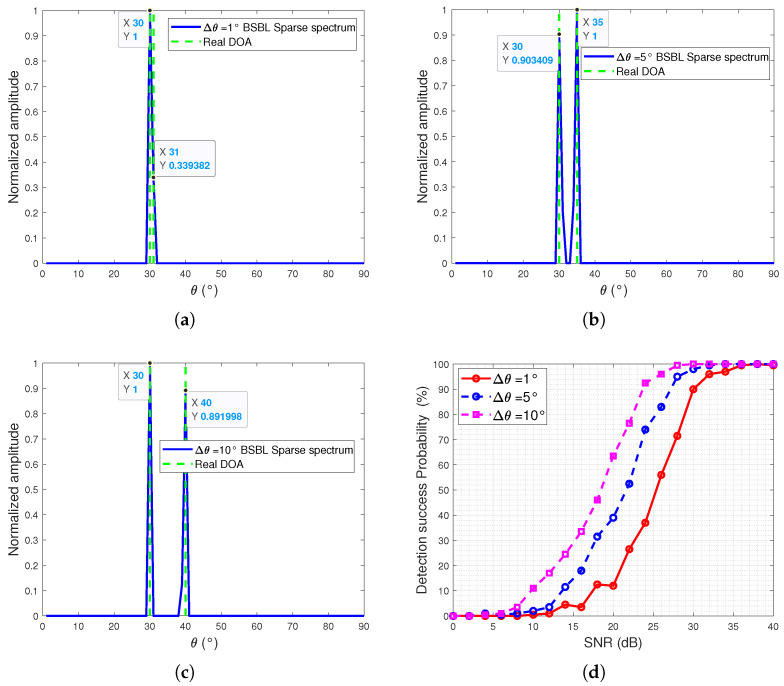
Estimating sparse spectrum with different intervals of DOA. (**a**) DOA estimation of sparse spectrum of $∆\theta =1^{\circ }$. (**b**) DOA estimation of sparse spectrum of $∆\theta =5^{\circ }$. (**c**) DOA estimation of sparse spectrum of $∆\theta =10^{\circ }$. (**d**) DOA estimation of detection success probability.

When the DOA intervals of multiple incoming signals are relatively close, the parameter estimation resolution of the traditional algorithm will decrease. Change the angular separation between the two coherent sources, and ∆θ is respectively $1^{\circ }$, $5^{\circ }$, and $10^{\circ }$. That is, in the three sets of experiments, the incident angles of the signals were $\left(30^{\circ },31^{\circ }\right)$, $\left(30^{\circ },35^{\circ }\right)$, and $\left(30^{\circ },40^{\circ }\right)$. The other conditions were the same as those in the simulation experiment in Section 4.1. The specific simulation results are shown in Figure 6.

In Figure 6, the improved block structure algorithm based on BSBL proposed in this paper exhibits distinct spectral peaks when the angle intervals of multiple signals are greater than $5^{\circ }$, enabling accurate discrimination of multiple coherent signals. However, when the angle interval is $1^{\circ }$, since the incident angles of the two coherent signals are too close, no obvious two spectral peaks appear in Figure 6a. Despite this, it can be seen that there are also two relatively large signal peaks in other spatial domains, which can distinguish these two coherent signals. Furthermore, as can be seen in Figure 6d, when the angle difference between the two signals increases, the signal resolution becomes higher. The main reason is that the decoupling processing of DOA and polarization parameters reduces the correlation interference of nearby angle sources, so that the angle information and polarization characteristics of each signal are respectively in the super-complete dictionary and the signal block. Even if the angle intervals of each source are small, the unique characteristics after parameter separation can still achieve effective signal separation. At the same time, the block structure design with real and imaginary part constraints enhances the sparsity feature of the signal, improving the accuracy and stability of sparse recovery. Therefore, the algorithm proposed in this paper is not sensitive to the angle interval of the incident angles of coherent signals and has super-resolution ability compared with traditional subspace-based algorithms.

### 4.3. Influence of Signal-to-Noise Ratio Parameter Estimation Performance

**Figure 7:**
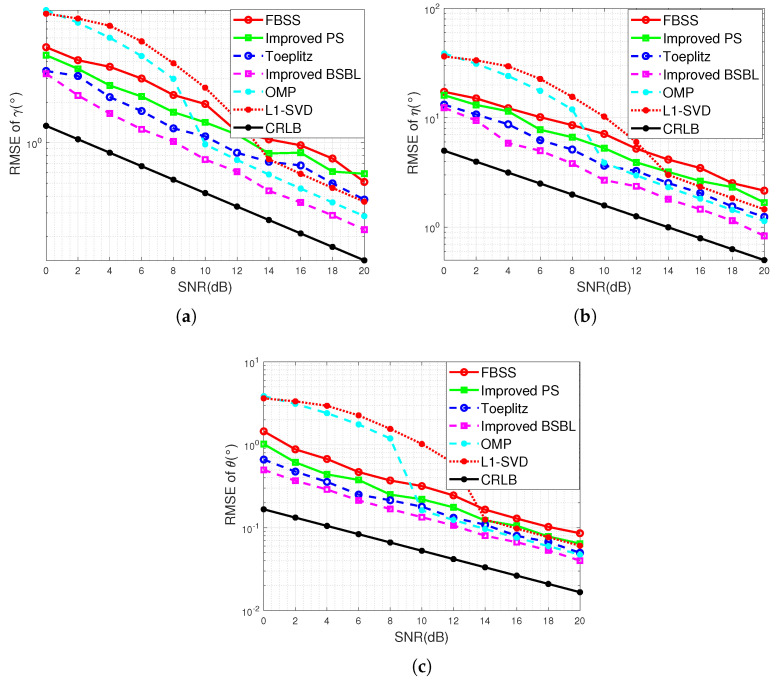
Influence of different SNR on RMSE performance with different algorithms. (**a**) RMSE of polarization parameter γ estimation. (**b**) RMSE of polarization parameter η estimation. (**c**) RMSE of polarization parameter θ estimation.

In Gaussian noise cases, we compared the RMSE performance of the FBSS algorithm, the improved PS algorithm, the Toeplitz algorithm, the L1-SVD algorithm, the OMP algorithm and the algorithm proposed in this paper in terms of parameter estimation as the SNR changed. RMSE is defined as

(32) $$RMSE_{P}=\sqrt{\frac{1}{T}\sum_{t=1}^{T}{\left({\hat{P}}_{t}-P\right)}^{2}}$$

where *P* represents parameters θ, γ, and η respectively, ${\hat{P}}_{t}$ represents the estimated values of these three parameters respectively. *T* is the number of Monte Carlo simulations. The incoming wave directions setting was $\left(\theta_{1},\gamma_{1},\eta_{1}\right)=\left(30^{\circ },60^{\circ },15^{\circ }\right),\;\left(\theta_{2},\gamma_{2},\eta_{2}\right)=\left(45^{\circ },45^{\circ },30^{\circ }\right)$, the SNR ranged from 0dB to 20dB, the grid point number was $\bar{K}=900$, and the other conditions were the same as those in the simulation experiment in Section 4.1, as shown in Figure 7.

In the simulation results shown in Figure 7, within the range of SNR from 0 dB to 20 dB, the root mean square error of parameter estimation for these de-coherence algorithms shows a decreasing trend as the SNR increases. The parameter estimation performance of the algorithm based on Toeplitz matrix reconstruction is superior to that of the FBSS algorithm and the polarization smoothing algorithm. The reason is that the estimation accuracy of the subspace-based algorithms highly depends on the estimation quality of the covariance matrix, and the covariance matrix obtained by Toeplitz matrix reconstruction is closer to the theoretical covariance matrix, thus resulting in better performance. The OMP algorithm and the L1-SVD algorithm have poorer parameter estimation performance compared to other algorithms within the range of 0 dB to 10 dB. However, their performance is comparable to that of the Toeplitz matrix reconstruction algorithm after the SNR exceeds 10 dB, indicating that these two algorithms have higher requirements for SNR. Meanwhile, the root mean square error of parameter estimation for the proposed algorithm in this paper decreases with the increase in SNR, which indicates that the proposed algorithm based on the improved block structure of BSBL is superior to the subspace-based parameter estimation algorithms, and the OMP algorithm when the SNR is lower than 20 dB. This is mainly because the proposed algorithm does not rely on the estimation of the signal covariance matrix, and the parameter decoupling strategy in the algorithm reduces the redundancy of the dictionary and improves the signal identification in noisy environments. Moreover, the block structure design based on real and imaginary part constraints strengthens the structured sparse features of the signal, thereby improving the performance of parameter estimation.

### 4.4. The DOA Estimation Performance of Different Snapshot Numbers

**Figure 8:**
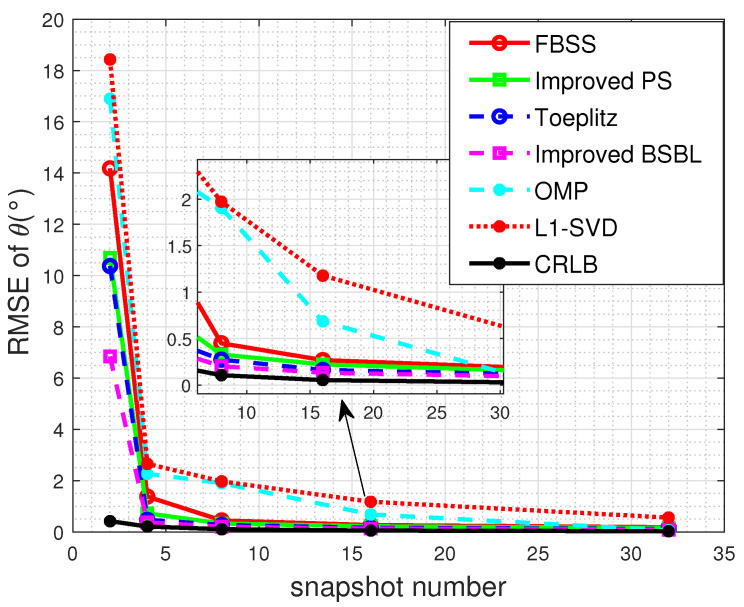
RMSE of θ with snapshot number.

In Gaussian noise cases, we compared the RMSE performance of the FBSS algorithm, the improved PS algorithm, the Toeplitz algorithm, the L1-SVD algorithm, the OMP algorithm and the algorithm proposed in this paper in terms of parameter estimation as the snapshot number changed. The SNR was fixed at 10 dB, and the number of sample snapshots was between 2 and 32, with the discrete angle set $\bar{\theta }=\left[1^{\circ }:0.5^{\circ }:90^{\circ }\right]$, and the other conditions were the same as those in the simulation experiment in Section 4.1, as shown in Figure 8.

As can be seen from Figure 8, as the number of sampling snapshots increases gradually from 2 to 32, the root mean square errors (RMSEs) of parameter estimation for all these decoherence algorithms show a continuous downward trend. When N=2, the RMSEs of all algorithms are greater than $5^{\circ }$; when N≥4, the RMSE of the proposed algorithm tends to be stable. This indicates that the proposed algorithm has a lower bound on the number of sampling snapshots, and it can achieve satisfactory performance only when N≥4.

In addition, the proposed algorithm exhibits better estimation performance in terms of RMSE under small snapshot conditions compared with subspace-based L1-SVD and OMP parameter estimation algorithms. The reason is that the sparse Bayesian framework constrains the sparsity of signals based on prior distributions. Under small snapshot conditions, such prior information is equivalent to adding effective constraints to the solution space, reducing the degrees of freedom of estimation, and enabling more accurate screening of the sparse components corresponding to the real sources with limited data, thus achieving performance optimization under small snapshot conditions. Furthermore, the design of the decoupled block model and the joint real-imaginary part constraint further enhances the parameter estimation performance compared with other comparative algorithms.

### 4.5. Performance of DOA Estimation with Off-Grid Model

**Figure 9:**
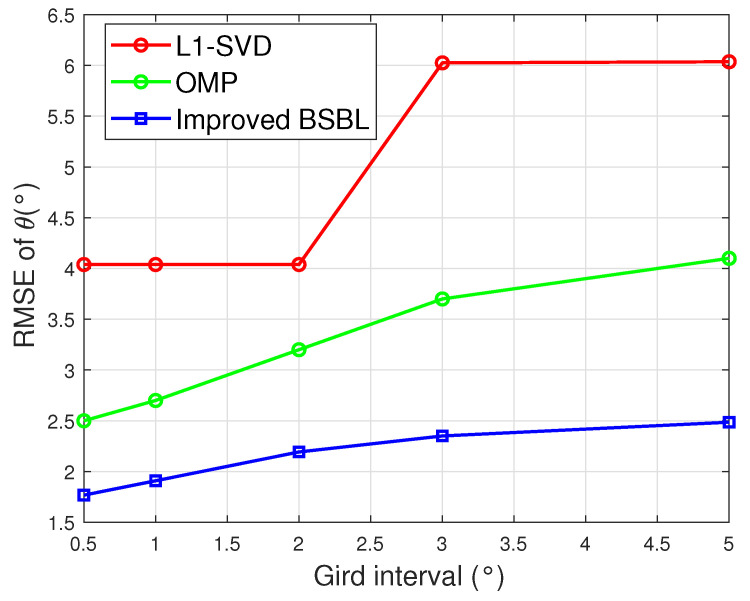
DOA estimation error for off-grid model.

In Gaussian noise cases, we compared the DOA estimation error of the OMP algorithm, the L1-SVD algorithm, and the proposed algorithm with off-grid intervals. The grid interval was set as $r=[0.5^{\circ },1^{\circ },2^{\circ },3^{\circ },5^{\circ }]$, the SNR was fixed at 10 dB, the number of snapshots was fixed at 16, and the discrete angle set $\bar{\theta }$ ranged from $0^{\circ }$ to $90^{\circ }$. The signal parameters were consistent with those in the simulation experiment in Section 4.1, and the number of Monte Carlo trials was set to 100. The simulation results are shown in Figure 9.

As can be seen from Figure 9, as the grid interval increases from $0.5^{\circ }$ to $5^{\circ }$, the DOA estimation RMSE of all three algorithms shows an upward trend. This is because a larger grid interval leads to a more significant deviation between the true DOA and the discrete grid points, making it impossible to accurately locate the true source position during sparse reconstruction, thereby introducing off-grid errors. The L1-SVD algorithm is the most sensitive to grid errors and exhibits the most significant performance degradation. The reason lies in that its $L_{1}$-norm constraint is highly dependent on the matching accuracy of dictionary atoms, and energy dispersion with coarse grids easily leads to peak ambiguity. The OMP algorithm alleviates energy leakage to a certain extent through greedy iterative atom-by-atom matching, resulting in moderate performance. The proposed BSBL-based improved block-structure algorithm enhances the sparsity characteristics of the signal through the block structure design with real and imaginary part constraints. It maintains a low RMSE for all grid intervals and demonstrates stronger robustness against grid interval errors.

### 4.6. Detection Success Probability of Parameter Estimation

**Figure 10:**
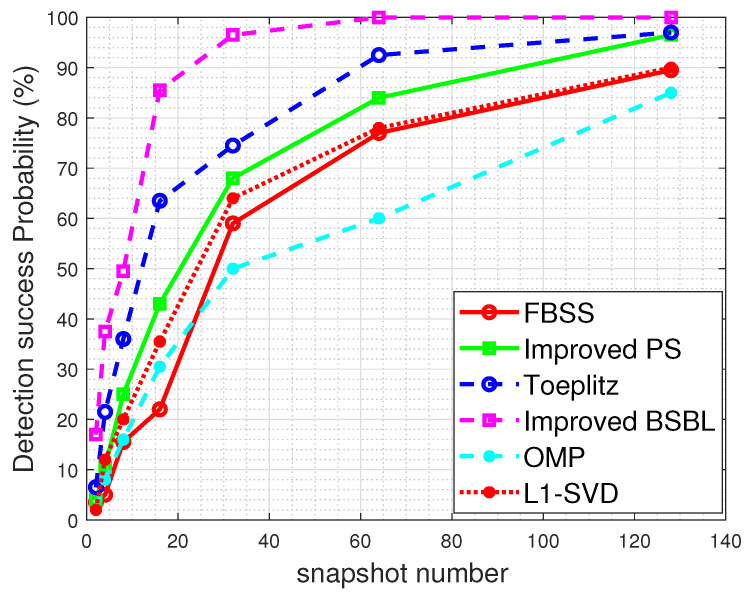
The detection success probability for different snapshot numbers.

In Gaussian noise cases, we compared the detection success probability of the FBSS algorithm, the improved PS algorithm, the Toeplitz algorithm, the L1-SVD algorithm, the OMP algorithm and the algorithm proposed in this paper as the snapshot number changed. The detection success rate here represents the number of successful detections in 100 independent experiments, where a successful detection in one experiment is defined as all parameter estimation results falling within the range of $\pm 0.5^{\circ }$ of the true values. The number of sampling snapshots varied from 2 to 128, with the discrete angle set $\bar{\theta }=\left[1^{\circ }:0.5^{\circ }:90^{\circ }\right]$, with other conditions identical to those in the simulation experiment in Section 4.1. The curve of detection rate versus the number of sample snapshots is shown in Figure 10.

As can be seen from Figure 10, all algorithms exhibit monotonically increasing detection success rates as the number of snapshots increases, which is attributed to the improved statistical reliability of parameter estimation with more abundant sampling data. The proposed improved BSBL algorithm achieves the best overall performance across the entire tested range of snapshot numbers. It demonstrates exceptional robustness in the extremely small snapshot regime, achieving approximately 40% detection success rate at only 4 snapshots and reaching 100% success rate at 60 snapshots, significantly outperforming all comparison algorithms. The reason for this is that the sparse Bayesian learning algorithm conducts statistical inference by integrating prior knowledge with sample data. The reduction in the number of snapshots has a smaller impact on the inference results compared to traditional subspace-based algorithms.

## 5. Conclusions

This paper addresses the challenging problem of joint direction of arrival (DOA) and polarization parameter estimation for coherent electromagnetic signals, which is critical for applications such as radar target recognition, electronic reconnaissance and wireless communication. Conventional subspace-based methods suffer from severe performance degradation under coherent signal conditions due to covariance matrix rank deficiency, while existing sparse reconstruction methods face issues of real-imaginary part misalignment, polarization information loss and unstable convergence in complex domains.

To overcome these limitations, we propose an improved block sparse Bayesian learning (BSBL) algorithm with a customized 4N-dimensional joint block structure. The core innovations and advantages include: (1) a Kronecker-product-based decoupling strategy that separates DOA and polarization information in the joint steering matrix, reducing dictionary redundancy and enhancing signal discriminability; (2) a unified block constraint that bundles the real and imaginary parts of both horizontal and vertical polarization components within the same block, inherently eliminating real-imaginary mismatch without additional post-processing; and (3) integration of these designs into the BSBL framework to leverage its inherent de-coherence capability and superior performance under limited data conditions. Simulation results show that the algorithm proposed in this paper has higher estimation accuracy under small snapshot conditions. Despite these advantages, the current algorithm still has some limitations. Its performance degrades slightly when incident signals deviate significantly from grid points, and computational complexity increases with grid density. Future work will focus on developing off-grid sparse Bayesian learning methods to eliminate grid mismatch effects, extending the algorithm to distributed dual-polarized arrays for 2D parameter estimation, and investigating robust estimation schemes in colored noise environments.

## Figures and Tables

**Table 1:** Comparison of computational complexity.

Algorithm Type	Total Computational Complexity
FBSS	$O\left({\left(2M\right)}^{2}N+{\left(2M\right)}^{3}+\bar{K}{\left(2M\right)}^{2}\right)$
Improved PS	$O\left({\left(2M\right)}^{2}N+{\left(2M\right)}^{3}+\bar{K}{\left(2M\right)}^{2}\right)$
Toeplitz	$O\left(M^{2}N+M^{3}+\bar{K}M^{2}\right)$
OMP	$O\left(K\bar{K}MN+{\bar{K}}^{3}\right)$
L1-SVD	$O\left({\left(2M\right)}^{3}+{\bar{K}}^{3}+\bar{K}{\left(2M\right)}^{2}\right)$
Improved BSBL	$O\left(4M\bar{K}N+I\cdot {\left(4NK\right)}^{3}+\bar{K}K\right)$

## Data Availability

The original contributions presented in this study are included in the article. Further inquiries can be directed to the corresponding author.
